# Volatile Flavor Compounds Profile and Fermentation Characteristics of Milk Fermented by *Lactobacillus delbrueckii* subsp. *bulgaricus*

**DOI:** 10.3389/fmicb.2019.02183

**Published:** 2019-09-18

**Authors:** Tong Dan, Weiyi Ren, Yang Liu, Jiale Tian, Haiyan Chen, Ting Li, Wenjun Liu

**Affiliations:** Key Laboratory of Dairy Biotechnology and Engineering, Dairy Processing Laboratory of National Dairy Production Technology and Research Center, Ministry of Education of the People’s Republic of China, Inner Mongolia Agricultural University, Hohhot, China

**Keywords:** fermented milk, *Lactobacillus delbrueckii* subsp. *bulgaricus*, SPME-GC-MS, volatile flavor compounds, fermentation characteristics

## Abstract

*Lactobacillus delbrueckii* subsp. *bulgaricus* is one of the predominant lactic acid bacterial species used as starter cultures in industrial fermented dairy manufacturing, as it strongly affects the quality of the products. Volatile flavor compound profiles and fermentation characteristics are considered to be the most important indicators for starter culture screening. In the present study, volatile compounds in milk fermented by 17 test strains of *L. delbrueckii* subsp. *bulgaricus* and a commercial strain used as a control were identified using solid-phase microextraction (SPME) methods coupled with gas chromatography mass spectrometry (GC-MS). In total, 86 volatile flavor compounds were identified in the fermented milk upon completion of fermentation, including 17 carboxylic acids, 14 aldehydes, 13 ketones, 29 alcohols, 8 esters, and 5 aromatic hydrocarbon compounds. Various volatile flavor compounds (acetaldehyde, 3-methyl-butanal, (E)-2-pentenal, hexanal, (E)-2-octenal, nonanal, 2,3-butanedione, acetoin, 2-heptanone, 2-non-anone, formic acid ethenyl ester) were identified due to their higher odor activity values (>1). In addition, of the 17 test strains of *L. delbrueck*ii subsp. *bulgaricus*, IMAU20312 (B14) and IMAU62081 (B16) strains exhibited good fermentation characteristics in milk compared with the control strain. The combination of the volatile flavor compound profile and fermentation characteristics in this work could be useful when selecting lactic acid bacteria that may serve as important resources in the development of novel fermented milk products.

## Introduction

Fermented dairy products have played an important role in the human diet for thousands of years. Notably, yogurt, of different names and forms, has constituted a vital component of the human diet in many regions around the world (Routray and Mishra, 2011). The yogurt aroma, involving the volatiles initially present in milk and compounds produced during fermentation and storage, is an important factor that contributes to yogurt quality (Ott et al., 2000; Cheng, 2010). Numerous investigations of flavor compounds have been published. Flavor composition is a complicated characteristic that involves a variety of volatile compounds, such as acids, aldehydes, ketones, alcohols, esters, and hydrocarbons (Valero et al., 2001; Dan et al., 2017). Thus far, more than 90 flavor compounds have been detected in fermented milk (Ott et al., 1997; Routray and Mishra, 2011). Solid-phase microextraction (SPME) coupled with gas chromatography mass spectrometry (GC-MS) can be used to detect these compounds associated with flavor. SPME is a simple and reliable technique for analysis of volatile compounds, allowing them to be effectively concentrated for analysis through GC-MS (Sattin et al., 2016; Stefanovic et al., 2018). Numerous recent SPME-GC-MS studies have investigated the odorant characteristics of yogurt (Settachaimongkon et al., 2014), cheese (Reale et al., 2016b; Ruggirello et al., 2018), fermented camel milk (Ning et al., 2011), and other fermented foods (Reale et al., 2016a; Di Renzo et al., 2018).

*Lactobacillus delbrueckii* subsp. *bulgaricus* is a non-pathogenic, lactic acid-producing, gram-positive rod bacteria that is frequently found in a variety of ecological niches, such as dairy foods (Batdorj et al., 2007; Karami et al., 2017), plants (Dawlal et al., 2019), and human intestine (Ren et al., 2014). In recent years, scientific understanding of the metabolism and functions of *L. delbrueckii* subsp. *bulgaricus* has increased considerably, thereby increasing the capacity of these bacteria to serve as starter cultures for industrial dairy applications (Giraffa et al., 2010). Some strains have been selected for use in fermented dairy products, such as fermented milk (Alférez et al., 2019), cheese (Silva et al., 2015), and frozen yogurt (Davidson et al., 2000).

Few studies have described the volatile composition and fermentation properties of milk fermented by *L. delbrueckii* subsp. *bulgaricus*. In the present study, the flavor profiles and physical properties of milk fermented by each of the 17 test strains of *L. delbrueckii* subsp. *bulgaricus* were compared with those of milk fermented under the same conditions using *L. delbrueckii* subsp. *bulgaricus* isolated from a commercial starter strain widely used in the dairy industry.

## Materials And Methods

### Strain Culture and Reagents

In total, 17 test strains of *L. delbrueckii* subsp. *bulgaricus* (14 from Mongolia, 1 from Xinjiang, and 2 from Tibetan China) were obtained from the Lactic Acid Bacteria Collection Center of Inner Mongolia Agricultural University, and cryopreserved (Table 1). *L. delbrueckii* subsp. *bulgaricus* TF-L904-b (a commercial yogurt starter culture, YF-L904; Chr. Hansen, Arpajon, France) was used as a control strain. All isolates were prepared in De Man, Rogosa and Sharpe (MRS) liquid media at 37°C for 24 h, and then inoculated into 50 and 500 mL MRS liquid medium for two generations at 37°C for 24 h. The resulting cells were collected and resuspended in phosphate-buffered saline (0.8% NaCl, 0.02% KCl, 0.02% KH_2_PO_4_, and 0.115% Na_2_HPO_4_) to prepare the bacterial suspension.

**TABLE 1:** Characteristics of 17 *L. delbrueckii* subsp. *bulgaricus* strains from traditional fermented milk in Mongolia, Xinjiang, and Tibetan China.

**No.**	**Strains**	**16S rRNA GeneBank**	**Isolation location**	**Dairy product**	**Fermentation time (h)**
C	TF-L904-b		Commercial starter cultures		10
B1	IMAU20402	HM058126	Khuvsgul province, Mongolia	Fermented cow milk	9
B2	IMAU20403	HM058127	Khuvsgul province, Mongolia	Fermented cow milk	10.2
B3	IMAU20360	HM058088	Bulgan Province, Mongolia	Fermented cow milk	8.7
B4	IMAU20364	HM058092	Bulgan Province, Mongolia	Fermented cow milk	13.8
B5	IMAU20396	HM058120	Bulgan Province, Mongolia	Fermented cow milk	10.2
B6	IMAU20220	HM057954	Dornod province, Mongolia	Fermented cow milk	8.2
B7	IMAU20227	HM057961	Dornod province, Mongolia	Fermented cow milk	10.2
B8	IMAU20228	HM057962	Dornod province, Mongolia	Fermented cow milk	17.8
B9	IMAU20234	HM057968	Dornod province, Mongolia	Fermented cow milk	9
B10	IMAU20769	HM058479	Wulaanbatar, Mongolia	Fermented cow milk	9.8
B11	IMAU20775	HM058485	Wulaanbatar, Mongolia	Fermented cow milk	9.5
B12	IMAU20790	HM058500	Wulaanbatar, Mongolia	Fermented cow milk	13.5
B13	IMAU20421	HM058145	Khuvsgul province, Mongolia	Fermented cow milk	8.7
B14	IMAU20312	HM058042	Eerhan province, Mongolia	Fermented cow milk	10
B15	IMAU32104	KF148790	Xinjiang province, China	Fermented cow milk	14.5
B16	IMAU62081	KF149723	Tibet, China	Fermented yak milk	9.3
B17	IMAU62115	KF149755	Tibet, China	Fermented yak milk	10.3

Standard *n*-alkanes (C3–C25) were obtained from AccuStandard (New Haven, CT, United States). An internal standard, 1,2-dichloro-benzene, was acquired from Sigma-Aldrich (Steinheim, Germany). MRS broth and whole milk powder were acquired from OXOID (Hampshire, England) and NZMP (Wellington, New Zealand), respectively.

### Fermented Milk Manufacturing

The procedure used for preparation of fermented milk was described previously (Dan et al., 2019). Briefly, 11.5% (w/v) whole milk powder was dissolved in distilled water supplemented with 6.5% sucrose. After consecutive homogenization procedures (65°C at 15 and 35 MPa, respectively) using a high-pressure homogenizer (Shanghai, China), the resulting milk was pasteurized (95°C, 5 min), rapidly cooled in ice water, and stored at 4°C until use.

Seventeen strains of *L. delbrueckii* subsp. *bulgaricus* and control strain were used to inoculate the milk at a concentration of 7.7 log_10_ CFU/mL; the inoculated milk was added to a 15-mL gas-phase flask and fermented in an incubator at 37°C. When the pH of the sample reached 4.5 and the titratable acidity (TA) reached 70–90°T, fermentation was ended quickly using an ice bath. The fermented milk was then maintained at 4°C while the volatile flavor compounds were determined. The pH value, TA, viable cell counts, and viscosity were analyzed at 3 and 6 h during fermentation, and at 0 and 12 h during refrigerated storage. All sample analyses were performed in triplicate.

### Fermentation Assays

#### pH and TA

The pH of the fermented milk was determined using a pHSJ-3F meter (Leici, Shanghai, China) at room temperature. TA was determined as previously described (Dan et al., 2019).

#### Viable Cell Counts

The numbers of viable cells of the 17 test strains of *L. delbrueckii* subsp. *bulgaricus* and the control strain in fermented milk samples were recorded by spread plating 100 μL of 10-fold serial dilutions on MRS agar, which was then incubated at 37°C for 48 h. Visible colonies were measured in log10 CFU/mL.

#### Viscosity

The viscosity of the fermented milk was measured in triplicate using a Brookfield DV-E Viscometer (Brookfield Engineering Laboratories, Middleboro, MA, United States), at a temperature of 20–22°C and speed of 100 rpm for 30 s.

### Determination of Volatile Flavor Compounds

#### Isolation of Volatile Flavor Compounds

Volatile flavor compounds were identified in accordance with a previously published method (Dan et al., 2018). Briefly, volatile flavor compounds were analyzed using a 7890B gas chromatograph equipped with a 5977A mass selective detector (Agilent Technologies, Inc., Palo Alto, CA, United States). SPME fibers (50/30 μm divinylbenzene/carboxen/polydimethylsiloxane; Supelco, Bellefonte, PA, United States) were reconditioned for 5 min at 250°C, then inserted above the gas-phase bottle for extraction for 60 min. Desorption was conducted at 250°C for 3 min.

The HP-5MS column (30 m length, 0.25 mm inside diameter, 0.25 μm film thickness; Agilent Technologies, Inc.) was initially held at 35°C, then heated to 140°C at a rate of 4°C/min, and finally heated to 250°C at a rate of 10°C/min, which was held for 3 min. The transfer line temperature was maintained at 250°C. Helium was used as the carrier gas with a flow rate of 1 mL/min, and no split sampling was used.

Mass spectrometry parameters included electron ionization at 70 eV, ion source temperature at 230°C, mass acquisition range (m/z) from 33 to 450, and emission current at 100 μA.

#### Qualitative Analysis

Volatile flavor compounds were identified using the National Institute of Standards Technology Mass Spectral Database 11 (accessed using software from Agilent Technologies, Inc.), based on comparison of mass spectra and retention indices (RIs). To calculate the RIs of the detected compounds, *n*-alkane standards (C3–C25) were analyzed under the same experimental conditions. An internal standard, 1,2-dichlorobenzene, was mixed in the samples.

#### Odor Activity Value

The odor activity value (OAV) was determined as previously described (Dan et al., 2019). In brief, the OAV was calculated as the ratio of the concentration of each compound in the sample relative to its previously reported sensory threshold concentration.

### Sensory Analysis

The flavors of the milk samples fermented by the 17 test strains of *L. delbrueckii* subsp. *bulgaricus*, and the control strain, at the end of fermentation were assessed by ten trained panelists, based on requirements specified in the Chinese dairy industry guideline RHB 103-2004. A beaker (100 mL) filled with each sample was used for evaluation. Flavor intensity was recorded on a scale ranging from 1 (strongly attractive) to 5 (strongly unappealing), and the total scores were determined.

### Statistical Analysis

Data were analyzed using Excel (Microsoft Corp., Redmond, WA, United States), SPSS, SIMCA-P (version 11.5; Umetrics AB, Umeå, Sweden), and SAS software (version 9.0; SAS Institute, Cary, NC, United States). These data included principal component analysis (PCA), significance tests, and correlation analysis. Origin (version 8.6; Microcal Software, Northampton, MA, United States) and heml version 1.0 were used to create histograms, line charts, principal component loading plots, score plots, and heat maps. Similarities were assessed on chromatograms obtained from the fermented milk samples using the Similarity Evaluation System for Chromatographic Fingerprint of Traditional Chinese Medicine (version A). All measurements were performed in triplicate.

## Results

### Physicochemical Characteristics

The fermentation times of the 17 test strains of *L. delbrueckii* subsp. *bulgaricus* are shown in Table 1. The fermentation times of nine strains (B1, B3, B6, B9, B10, B11, B13, B14, and B16) were less than that of the control strain (10 h); within 10 h, the pH of the samples had fallen below 4.5 or the TA had reached 80°T when fermented by these isolates.

Changes in pH were observed in milk during fermentation by each of the 17 test strains of *L. delbrueckii* subsp. *bulgaricus* and the control strain (Table 2). After 3 h of fermentation, the pH of the milk began to decrease rapidly, reaching approximately 4.5 upon completion of fermentation. Notably, the pH of the milk fermented by strain B6 reached 4.23 upon completion of fermentation, due to its capacity for rapid acidification. Unlike pH, increased TA was observed in milk during fermentation by all test strains of *L. delbrueckii* subsp. *bulgaricus* and the control strain. By the end of fermentation, the TA value of milk fermented by the control strain had reached 88.07°T. The TA values of milk fermented by two test strains (B6 and B16, 95.9 and 104.71°T, respectively) were higher than that of milk fermented by the control strain. The TA values of milk fermented by seven strains (B1, B3, B5, B8, B9, B13, and B14) were similar to that of milk fermented by the control strain.

**TABLE 2:** Characteristics of milk fermented by the 17 test strains of *L. delbrueckii* subsp. *bulgaricus*, and the control strain, during the course of fermentation (3 h, 6 h) and storage (0 day, 12 h).

**No.**	**pH**				**Titratable acidity (°T)**				**Viable counts (Log_10_ CFU/mL)**				**Viscosity (mPa s)**			
	**3 h (F)**	**6 h (F)**	**0 day (S)**	**12 h (S)**	**3 h (F)**	**6 h (F)**	**0 day (S)**	**12 h (S)**	**3 h (F)**	**6 h (F)**	**0 day (S)**	**12 h (S)**	**3 h (F)**	**6 h (F)**	**0 day (S)**	**12 h (S)**
C	6.2 ± 0.02^abc^	5.33 ± 0.01^b^	4.46 ± 0.00^c^	3.96 ± 0.01^e^	26.91 ± 2.82^i^	49.91 ± 2.82^g^	88.07 ± 1.41^a^	112.54 ± 2.82^b^	8.00 ± 0.4^cde^	8.71 ± 0.3^a^	8.85 ± 0.5^ab^	8.80 ± 0.4^a^	318 ± 2.11^ab^	330 ± 1.31^def^	750 ± 1.92^d^	1124 ± 3.2^b^
B1	5.91 ± 0.00^bcd^	4.94 ± 0.01^cdefg^	4.45 ± 0.01^c^	4.18 ± 0.01^d^	35.23 ± 1.41^e^	83.18 ± 2.82^a^	85.13 ± 1.41^c^	119.39 ± 1.41^a^	8.50 ± 0.3^cde^	8.57 ± 0.4^c^	8.85 ± 0.2^gh^	8.79 ± 0.2^j^	312 ± 1.37^ab^	382 ± 1.36^cd^	466 ± 3.72^g^	1113 ± 4.82^cde^
B2	5.88 ± 0.01^bcd^	5.1 ± 0.01^bcd^	4.46 ± 0.00^c^	4.37 ± 0.02^abc^	32.29 ± 1.41^gh^	59.69 ± 1.41^e^	74.37 ± 1.41^g^	82.20 ± 1.41^i^	8.30 ± 0.4^b^	8.30 ± 0.2^ef^	8.80 ± 0.3^fgh^	8.77 ± 0.2^ij^	231 ± 3.79^cde^	374 ± 3.89^cde^	704 ± 2.10^b^	799 ± 2.69^de^
B3	5.89 ± 0.00^bcd^	5.02 ± 0.02^cde^	4.45 ± 0.01^c^	4.27 ± 0.02^cd^	50.89 ± 0.00^b^	73.39 ± 1.41^c^	82.20 ± 1.41^cde^	112.54 ± 2.82^b^	8.18 ± 0.2^bc^	8.19 ± 0.5^fg^	8.47 ± 0.5^cde^	8.25 ± 0.2^gh^	278 ± 1.21^bc^	498 ± 1.21^a^	544 ± 2.47^fg^	716 ± 2.98^de^
B4	5.92 ± 0.01^bcd^	5.31 ± 0.00^b^	4.51 ± 0.02^c^	4.45 ± 0.01^ab^	34.25 ± 1.41^ef^	46.97 ± 1.41^h^	67.52 ± 2.82^hi^	84.65 ± 1.41^i^	7.86 ± 0.5^e^	8.20 ± 0.2^fg^	8.50 ± 0.3^gh^	8.30 ± 0.5^hij^	162 ± 1.04^fgh^	386 ± 2.17^cd^	676 ± 1.47^d^	618 ± 2.93^bcd^
B5	5.9 ± 0.00^bcd^	5.16 ± 0.01^bc^	4.48 ± 0.00^c^	4.36 ± 0.01^abc^	31.31 ± 1.41^h^	55.78 ± 2.82^f^	82.20 ± 1.41^cde^	92.96 ± 1.41^fg^	8.30 ± 0.2^cde^	8.45 ± 0.2^cde^	8.58 ± 0.2^cd^	8.57 ± 0.3^bc^	194 ± 1.98^efg^	300 ± 1.53^fg^	638 ± 2.13^ef^	764 ± 1.39^e^
B6	5.87 ± 0.00^bcd^	4.69 ± 0.02^g^	4.23 ± 0.02^d^	4.17 ± 0.01^d^	35.23 ± 1.41^e^	80.24 ± 1.41^b^	95.90 ± 1.41^a^	102.75 ± 2.82^d^	8.31 ± 0.2^cde^	8.76 ± 0.2^a^	8.82 ± 0.3^gh^	8.48 ± 0.4^cd^	360 ± 2.84^a^	464 ± ± 2.11^ab^	555 ± 3.72^fg^	964 ± 2.22^bc^
B7	5.45 ± 0.00^de^	4.96 ± 0.00^cdef^	4.44 ± 0.00^c^	4.35 ± 0.00^abc^	46.97 ± 2.82^c^	80.24 ± 2.82^b^	78.29 ± 1.41^defg^	113.51 ± 2.82^b^	7.98 ± 0.4^cde^	8.50 ± 0.2^cd^	8.58 ± 0.3^cde^	8.56 ± 0.2^defg^	150 ± 2.18^gh^	292 ± 2.91^fg^	734 ± 1.74^de^	790 ± 3.26^cde^
B8	6.28 ± 0.02^abc^	6.07 ± 0.00^a^	4.52 ± 0.01^c^	4.36 ± 0.00^abc^	26.91 ± 1.41^i^	29.36 ± 1.41^j^	83.18 ± 1.41^cd^	88.07 ± 1.41^h^	8.12 ± 0.4^cd^	8.45 ± 0.2^cde^	8.85 ± 0.5^a^	8.48 ± 0.2^cd^	178 ± 2.67^h^	208 ± 3.88^hi^	1207 ± 3.73^a^	1805 ± 1.08^a^
B9	5.42 ± 0.00^de^	4.88 ± 0.01^defg^	4.44 ± 0.00^c^	4.33 ± 0.01^abc^	46.97 ± 1.41^c^	67.52 ± 1.41^d^	84.16 ± 1.41^cd^	91.01 ± 1.41^g^	7.98 ± 0.3^cde^	8.17 ± 0.3^fg^	8.17 ± 0.2^fgh^	7.95 ± 0.3^j^	212 ± 2.25^ef^	368 ± 2.26^cde^	485 ± 1.69^g^	775 ± 2.23^de^
B10	5.75 ± 0.01^cde^	5.13 ± 0.00^bcd^	4.62 ± 0.00^b^	4.34 ± 0.01^abc^	34.25 ± 1.41^ef^	66.54 ± 1.41^d^	76.33 ± 2.82^efg^	96.88 ± 2.82^e^	7.95 ± 0.2^de^	8.56 ± 0.5^c^	8.74 ± 0.2^cde^	8.56 ± 0.2^def^	210 ± 1.85^ef^	314 ± 1.86^efg^	634 ± 2.76^ef^	1079 ± 3.45^b^
B11	5.57 ± 0.00^de^	4.97 ± 0.00^cdef^	4.46 ± 0.02^c^	4.46 ± 0.01^a^	43.06 ± 1.41^d^	74.37 ± 1.41^c^	75.35 ± 1.41^fg^	106.18 ± 1.41^c^	7.94 ± 0.2^de^	8.52 ± 0.4^cd^	8.66 ± 0.2^def^	8.55 ± 0.2^ghi^	192 ± 2.08^gh^	270 ± 5.73^fgh^	574 ± 2.03^de^	664 ± 1.53^e^
B12	5.6 ± 0.00^de^	5.31 ± 0.00^b^	4.51 ± 0.01^c^	4.41 ± 0.00^abc^	43.06 ± 1.41^d^	58.71 ± 2.82^e^	73.39 ± 1.41^gh^	90.03 ± 2.82^gh^	8.43 ± 0.2^a^	8.56 ± 0.2^c^	8.69 ± 0.5^efg^	8.58 ± 0.3^gh^	342 ± 1.53^a^	478 ± 1.87^ab^	884 ± 3.74^c^	889 ± 1.63^bcd^
B13	5.3 ± 0.01^e^	4.76 ± 0.00^fg^	4.44 ± 0.01^c^	4.35 ± 0.00^abc^	53.82 ± 1.41^a^	74.37 ± 1.41^c^	87.09 ± 2.82^c^	91.99 ± 2.82^g^	7.88 ± 0.5^de^	8.58 ± 0.2^bc^	8.55 ± 0.2^h^	8.47 ± 0.3^de^	322 ± 1.17^ab^	432 ± 1.66^bc^	551 ± 3.28^fg^	698 ± 1.29^de^
B14	6.31 ± 0.00^ab^	5.01 ± 0.00^cdef^	4.45 ± 0.02^c^	4.27 ± 0.01^cd^	25.44 ± 2.82^i^	39.14 ± 1.41^i^	81.22 ± 1.41^cdef^	94.92 ± 1.41^ef^	8.27 ± 0.3^cde^	8.62 ± 0.2^def^	8.64 ± 0.3^ef^	8.55 ± 0.5^efg^	214 ± 1.51^def^	232 ± 1.46^hi^	670 ± 2.37^de^	916 ± 2.27^bcd^
B15	5.8 ± 0.02^bcde^	4.88 ± 0.01^efg^	4.44 ± 0.01^c^	4.31 ± 0.00^abcd^	26.42 ± 1.41^i^	26.91 ± 1.41^j^	63.61 ± 2.82^i^	92.96 ± 1.41^fg^	8.12 ± 0.4^cde^	8.17 ± 0.2^g^	8.59 ± 0.2^c^	8.63 ± 0.2^b^	268 ± 1.34^bcd^	372 ± 1.27^i^	456 ± 2.15^g^	420 ± 1.57^f^
B16	6.06 ± 0.01^a^	4.95 ± 0.01^a^	4.45 ± 0.01^a^	4.29 ± 0.00^abc^	23.98 ± 1.41^j^	45.99 ± 2.82^h^	104.71 ± 2.82^a^	105.69 ± 2.82^c^	8.03 ± 0.2^cde^	8.67 ± 0.2^ab^	8.78 ± 0.2^b^	8.75 ± 0.3^a^	178 ± 1.57^efg^	292 ± 5.28^fg^	312 ± 1.74^h^	768 ± 2.01^cde^
B17	5.93 ± 0.00^bcd^	4.91 ± 0.01^cdefg^	4.44 ± 0.02^c^	4.3 ± 0.02^bcd^	33.27 ± 2.82^fg^	59.69 ± 2.82^e^	72.41 ± 1.41^gh^	91.01 ± 1.41^g^	8.23 ± 0.2e	8.13 ± 0.3^fg^	8.31 ± 0.2^gh^	8.09 ± 0.3^j^	148 ± 2.46^gh^	252 ± 2.78^gh^	638 ± 3.51^ef^	782 ± 3.04^cde^

*F: fermentation; S: storage. Values within a column followed by different lowercase letters are significantly different at *P* < 0.05.*

Changes were observed in viable cell counts in milk fermented by each of the 17 test strains of *L. delbrueckii* subsp. *bulgaricus* and the control strain (Table 2). The viable cell count in milk fermented by the control strain increased rapidly during fermentation, reaching 8.85 log_10_ CFU/mL upon completion of fermentation. Similar results were found in milk fermented by the B1, B2, B6, B8, B10, B11, B12, B14 and B16 strains, which had viable cell counts close to 8.85 log_10_ CFU/mL upon completion of fermentation.

During fermentation, the viscosity of the milk fermented by the control strain increased significantly over time and peaked at 750 mPa s upon completion of fermentation. Similarly, viscosity increased steadily in milk fermented by strains B2, B4, B5, B7, B8, B10, B12, B14, and B17, reaching 704, 676, 638, 734, 1207, 634, 884, 670, and 638 mPa s, respectively, upon completion of fermentation. Samples of milk fermented by the remaining eight test strains (B1, B3, B6, B9, B11, B13, B15, and B16) had comparatively lower viscosity.

### Volatile Flavor Compounds in Fermented Milk

To evaluate the aroma profiles of milk fermented by each of the 17 test strains of *L. delbrueckii* subsp. *bulgaricus* and the control strain, the SPME-GC-MS technique was used to analyze the volatile compound composition of samples upon completion of fermentation (Supplementary Table S1). In total, 86 volatile flavor compounds were identified, including various types of carboxylic acids (17), aldehydes (14), ketones (13), alcohols (29), esters (8), and aromatic hydrocarbons (5). These compounds were identified by MS and their RIs, as determined by the HP-5MS column, were compared with those reported in the literature.

**FIGURE 1:**
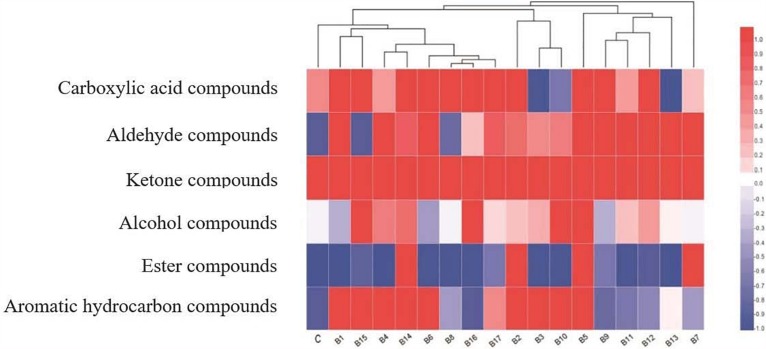
Heat map and dendrogram of volatile flavor compound profiles of samples fermented by 17 test strains of *L. delbrueckii* subsp. *bulgaricus*, and the control strain, upon completion of fermentation.

Samples fermented by the 17 test strains of *L. delbrueckii* subsp. *bulgaricus*, and the control strain, could be clearly assigned to different clusters (Figure 1). Furthermore, the samples fermented by strains B1, B4, B6, B8, B14, B15, B16, and B17, and the control strain, could be clearly grouped based on their volatile flavor compound profiles. The major aroma volatile compounds were ketones, followed by carboxylic acids; trace concentrations of alcohol compounds were also detected in milk fermented by the control strain. Ketone compounds showed high relative abundance (Figure 1, red areas) in samples fermented by the 17 test strains of *L. delbrueckii* subsp. *bulgaricus* and the control strain. Carboxylic acid compounds were present in all samples, except those fermented by strains B3, B10, and B13. Among the above-mentioned compounds, there were considerable differences in alcohol compound content. Higher levels of alcohol compounds were found in samples fermented by strains B5, B10, B15, and B16.

### Key Volatile Compounds in Fermented Milk

Volatile compounds can be potent odorants of sample aromas only when their concentrations in such samples exceed their sensory threshold concentrations (Majcher and Jelen, 2011). Therefore, the OAV of each volatile compound was calculated as a ratio of its concentration to the odor threshold. The OAVs of partial volatile compounds in milk fermented by the 17 test strains of *L. delbrueckii* subsp. *bulgaricus*, and the control strain, upon completion of fermentation are summarized in Table 3. Eleven compounds (acetaldehyde, 3-methyl-butanal, (E)-2-pentenal, hexanal, (E)-2-octenal, nonanal, 2,3-butanedione, acetoin, 2-heptanone, 2-non-anone, and ethenyl ester formic acid) with OAVs ≥ 1 are key contributors to the flavor of fermented milk. The key volatile compounds in milk fermented by the control strain were 2,3-butanedione, acetoin, 2-heptanone, and 2-non-anone. In particular, the OAV for 2-heptanone was 20.58, which indicated that this compound could be a significant contributor to the flavor of the sample. Similar results were found in samples of milk fermented by strains B14 and B16: 2-heptanone had OAVs of 30.35 and 12.73, respectively. In addition, 3-methyl-butanoic acid and 1-heptanol, with OAVs of 0.1–1, can modify the integral flavor of the samples.

**TABLE 3:** Odor activity values (OAVs) of volatile flavor compounds produced in milk fermented by 17 test strains of *L. delbrueckii* subsp. *bulgaricus*, and the control, strain upon completion of fermentation.

**Volatile compound**	**Odor threshold (μg/L)**	**Odor description**	**OAV**																		**References**
			
			**C**	**B1**	**B2**	**B3**	**B4**	**B5**	**B6**	**B7**	**B8**	**B9**	**B10**	**B11**	**B12**	**B13**	**B14**	**B15**	**B16**	**B17**	

Butanoic acid, 3-methyl-	130	rancid, cheesy, sweaty, putrid	−	0.22	0.33	−	0.02	0.80	0.38	0.04	0.13	0.06	0.04	0.06	0.13	−	0.13	0.31	0.40	0.20	Leffingwell and Leffingwell, 1991; Curioni and Bosset, 2002
Acetaldehyde	9	Ethereal, fresh, green, pungent	−	2.59	−	1.33	3.98	8.28	4.41	5.04	−	2.20	2.02	2.97	1.04	2.72	3.63	−	1.94	2.17	Sun, 2003; Ning et al., 2011
Butanal, 3-methyl-	5.4	Green, malty, unripe, cocoa, malty	−	1.69	3.33	2.24	1.76	2.85	1.44	2.24	0.42	2.08	1.73	1.36	2.89	1.59	1.18	0.33	0.44	0.82	Curioni and Bosset, 2002
2-Pentenal, (E)	1.2		−	−	−	−	−	1.85	−	−	−	−	−	−	−	−	−	−	2.78	−	Qian and Reineccius, 2003; Sun, 2003
Hexanal	3	Green, cut-grass	−	2.29	1.15	0.73	0.94	2.08	−	0.92	0.12	0.62	0.67	2.97	11.15	2.26	−	−	1.08	1.13	Curioni and Bosset, 2002; Gemert, 2003
2-Octenal, (E)-	3	Cucumber, fresh, green, waxy	−	−	−	−	−	−	−	−	−	−	−	−	−	−	−	−	−	1.32	John, 2001; Ning et al., 2011
Non-anal	1	Green, fatty, tallowy	−	−	−	1.39	0.59	−	−	−	−	0.82	0.53	0.79	−	0.65	−	0.50	0.53	1.25	Curioni and Bosset, 2002; Gemert, 2003
2,3-Butanedione	10	Buttery	8.36	−	−	−	−	−	−	−	−	−	−	−	−	−	6.33	−	5.07	−	Curioni and Bosset, 2002; Qian and Reineccius, 2003
Acetoin	55	Buttery	1.46	0.87	1.27	−	1.09	1.36	1.80	0.25	0.52	0.21	0.21	0.16	−	−	2.66	1.93	2.52	1.00	Curioni and Bosset, 2002; Qian and Reineccius, 2003
2-Heptanone	5	Fruity, fatty, Green	20.58	13.23	15.12	8.07	9.37	15.98	7.95	11.05	7.29	8.04	7.93	12.79	11.44	9.87	30.35	6.77	12.73	8.75	Curioni and Bosset, 2002; Attaie, 2009
2-Non-anone	5	Malty, fruity	11.93	5.49	5.59	2.63	2.97	7.28	3.63	3.64	3.37	3.00	2.80	4.57	3.56	2.74	13.10	2.19	5.52	2.97	Curioni and Bosset, 2002; Attaie, 2009
Heptanol	41	A fragrant, woody, heavy, oily, faint, aromatic, fatty	0.16	0.18	0.23	0.15	0.28	0.21	0.16	0.21	0.13	0.15	0.10	0.22	0.28	0.15	0.25	0.16	0.20	0.20	Gemert, 2003; Ning et al., 2011
Formic acid, ethenyl ester	9		−	0.14	11.48	−	−	10.11	−	12.42	−	−	−	−	−	−	7.82	−	−	1.40	Gemert, 2003
																					

### PCA of Volatile Compounds

**FIGURE 2:**
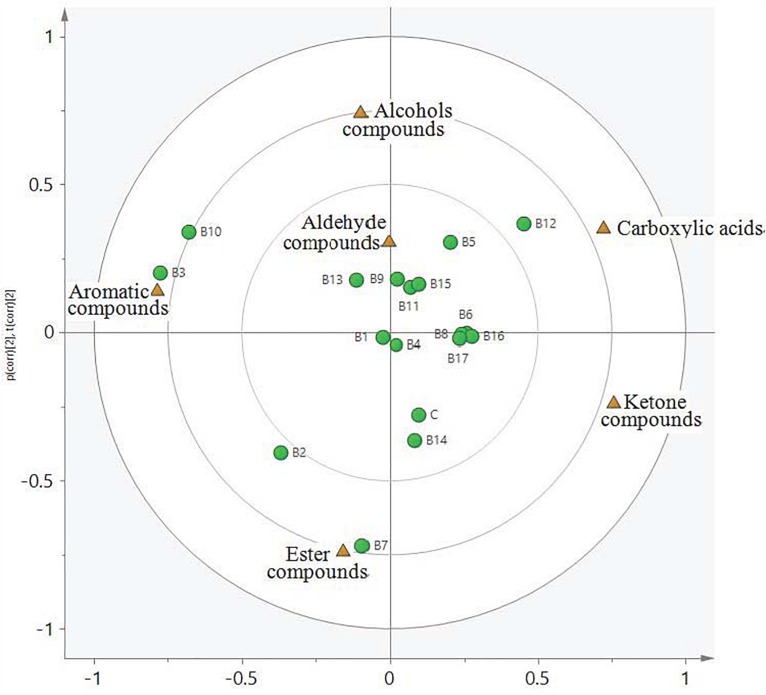
Loading plot and score plot from principal component analysis of all *L. delbrueckii* subsp. *bulgaricus* samples at the completion of fermentation. Triangular and circular symbols represent flavor compounds and strains, respectively.

Principal component analysis uses a few characteristics (i.e., principal components) to explain internal relationships among multiple covariates, thereby allowing objective assessment of the types and contents of substances in experimental samples. Aldehydes, ketones, carboxylic acids, esters, alcohols, and aromatic hydrocarbons in all samples were divided into four categories, or quadrants (Figure 2). All compounds were clearly distinct from strains and other compounds. There were higher correlations between aldehyde compounds and strains B9, B11, B12, B13, and B15. Similar correlations were found between ketone compounds and the control strain, as well as strains B1, B4, B6, B8, B14, B16, and B17. Carboxylic acid compounds were correlated with the B12 strain, alcohol compounds were correlated with the B5 strain, and aromatic hydrocarbon compounds were correlated with the B2, B3, and B10 strains.

### Similarity Analysis

The fingerprint data for the samples fermented by the 17 test strains of *L. delbrueckii* subsp. *bulgaricus*, and the control strain, were used to analyze similarities among the samples, by comparing each chromatogram with a reference chromatogram (Table 4). Similarity values among the samples fermented by the 17 test strains of *L. delbrueckii* subsp. *bulgaricus*, and the control strain, were in the range of 0.294–0.758. Among them, similarity values between samples fermented by strains B4, B12, B14, B16 and the control strain were >0.6, which indicated that there were minimal differences between samples fermented by these strains and those fermented by the control strain with respect to flavor compounds.

**TABLE 4:** Similarities of the chromatograms obtained from milk fermented by 17 test strains of *L. delbrueckii* subsp. *bulgaricus*, and the control strain, upon completion of fermentation.

	**C**	**B1**	**B2**	**B3**	**B4**	**B5**	**B6**	**B7**	**B8**	**B9**	**B10**	**B11**	**B12**	**B13**	**B14**	**B15**	**B16**	**B17**	**References**
C	1	0.457	0.57	0.412	0.673	0.564	0.582	0.57	0.47	0.325	0.373	0.589	0.641	0.311	0.743	0.334	0.758	0.294	0.809
B1	0.457	1	0.444	0.838	0.282	0.449	0.359	0.607	0.548	0.769	0.614	0.535	0.567	0.732	0.47	0.411	0.347	0.798	0.653
B2	0.57	0.444	1	0.281	0.582	0.666	0.511	0.436	0.256	0.499	0.208	0.459	0.472	0.437	0.599	0.173	0.596	0.333	0.717
B3	0.412	0.838	0.281	1	0.331	0.342	0.307	0.528	0.592	0.633	0.512	0.509	0.606	0.832	0.461	0.384	0.354	0.787	0.604
B4	0.673	0.282	0.582	0.331	1	0.558	0.859	0.712	0.257	0.198	0.251	0.796	0.558	0.33	0.704	0.179	0.788	0.208	0.816
B5	0.564	0.449	0.666	0.342	0.558	1	0.672	0.568	0.338	0.521	0.512	0.522	0.656	0.391	0.556	0.598	0.606	0.327	0.796
B6	0.582	0.359	0.511	0.307	0.859	0.672	1	0.836	0.244	0.372	0.401	0.877	0.481	0.358	0.59	0.336	0.649	0.281	0.814
B7	0.57	0.607	0.436	0.528	0.712	0.568	0.836	1	0.46	0.587	0.65	0.938	0.638	0.521	0.584	0.42	0.576	0.435	0.828
B8	0.47	0.548	0.256	0.592	0.257	0.338	0.244	0.46	1	0.401	0.558	0.431	0.633	0.489	0.408	0.595	0.345	0.461	0.549
B9	0.325	0.769	0.499	0.633	0.198	0.521	0.372	0.587	0.401	1	0.728	0.452	0.409	0.776	0.319	0.405	0.237	0.627	0.579
B10	0.373	0.614	0.208	0.512	0.251	0.512	0.401	0.65	0.558	0.728	1	0.5	0.571	0.499	0.348	0.581	0.293	0.435	0.58
B11	0.589	0.535	0.459	0.509	0.796	0.522	0.877	0.938	0.431	0.452	0.5	1	0.624	0.506	0.612	0.352	0.594	0.394	0.826
B12	0.641	0.567	0.472	0.606	0.558	0.656	0.481	0.638	0.633	0.409	0.571	0.624	1	0.502	0.699	0.586	0.68	0.407	0.808
B13	0.311	0.732	0.437	0.832	0.33	0.391	0.358	0.521	0.489	0.776	0.499	0.506	0.502	1	0.388	0.338	0.268	0.68	0.581
B14	0.743	0.47	0.599	0.461	0.704	0.556	0.59	0.584	0.408	0.319	0.348	0.612	0.699	0.388	1	0.287	0.797	0.301	0.845
B15	0.334	0.411	0.173	0.384	0.179	0.598	0.336	0.42	0.595	0.405	0.581	0.352	0.586	0.338	0.287	1	0.3	0.336	0.509
B16	0.758	0.347	0.596	0.354	0.788	0.606	0.649	0.576	0.345	0.237	0.293	0.594	0.68	0.268	0.797	0.3	1	0.264	0.833
B17	0.294	0.798	0.333	0.787	0.208	0.327	0.281	0.435	0.461	0.627	0.435	0.394	0.407	0.68	0.301	0.336	0.264	1	0.503
Reference	0.809	0.653	0.717	0.604	0.816	0.796	0.814	0.828	0.549	0.579	0.58	0.826	0.808	0.581	0.845	0.509	0.833	0.503	1

### Sensory Assessment

Flavor evaluations of samples fermented by the 17 test strains of *L. delbrueckii* subsp. *bulgaricus*, and the control strain, were performed by ten trained panelists at the end of the fermentation. The scores of the B14 and B16 samples were better than samples fermented by the other strains (data not shown).

## Discussion

Fermentation time, pH, TA, and viable cell counts are regarded as important factors for the selection of starter cultures with the best fermentation characteristics (Rault et al., 2009). In this study, the fermentation times of the 17 test strains of *L. delbrueckii* subsp. *bulgaricus* and the control strain ranged from 8.2 to 17.8 h. The fermentation times of strains B1, B3, B6, B9, B10, B11, B13, B14, and B16 were shorter than that of the control strain (10 h), indicating that these strains have robust acid-production properties in milk.

pH and TA values are key indicators of the acidity of fermented milk, reflecting the characteristics of acid-production dynamics (Liu et al., 2016). In the present study, the pH and TA values in milk samples fermented by strains B1, B3, B5, B8, B9, B13, and B14 were similar to those of milk fermented by the control strain during the course of fermentation (3 h, 6 h) and storage (0 day, 12 days). The results indicated that these strains had higher acidification efficiency than the remaining ten test strains. Sugar utilization tests indicate that some *L. delbrueckii* subsp. *bulgaricus* can metabolize sucrose (Chick et al., 2001). In the present study, 6.5% sucrose was added to milk, which could be one reason for the observed rapid decrease in pH and growth of lactic acid bacteria in milk.

Viable cell counts are very important to the final products of the dairy industry. Dairy products containing lactic acid bacteria confer health benefits to humans when they accumulate a sufficient amount of bacteria (Shori, 2013). Viable cell counts of samples fermented by 17 test strains of *L. delbrueckii* subsp. *bulgaricus* and the control strain gradually increased during fermentation (Table 2), and no consistent differences in viable counts were found among samples upon completion of fermentation. Viable cell counts of samples fermented by strains B1, B2, B6, B8, B10, B11, B12, B14 and B16 approached 8.85 log_10_ CFU/mL (control strain sample) upon completion of fermentation and was maintained at 8.48 log_10_ CFU/mL during storage (0 day, 12 days). Similar results were reported by Naji et al. (2014). The viscosity of the samples fermented by strains B2, B4, B5, B7, B8, B10, B12, B14, and B17 was similar to or exceeded that of the sample fermented by the control strain upon completion of fermentation. This was consistent with the findings of Shihata and Shah (2002), who showed that fermented milk samples inoculated with extremely high levels of *L. delbrueckii* subsp. *bulgaricus* had higher apparent viscosity. In addition, different increases were observed in the viscosity of milk fermented by the 17 test strains of *L. delbrueckii* subsp. *bulgaricus*. These differences may be due to the fermentation process used by lactic acid bacteria in milk products to synthesize exopolysaccharides, which play an important role as natural bio-thickening agents, increasing the viscosity of fermented milk (Han et al., 2016).

Carboxylic acid compounds are major components of most dairy products. These compounds are produced during yogurt fermentation by lipolytic processes and bacterial fermentation. Acetic acid is an important compound that can provide a “vinegary” taste to dairy products (Cheng, 2010). Acetic acid was detected in samples B1, B4, B5, B6, B7, B8, B10, B12, B14, B15, B16, and B17 in this study; in samples fermented by strains B5, B6, B15, and B16, concentrations of acetic acid were higher than that in the sample fermented by the control strain (12.48 μg/L). Hexanoic acid provides a “cheesy,” “rancid,” and “sweat-like” flavor to dairy products (Condursoa et al., 2008). Compared with the level in the sample fermented by the control strain (11.52 μg/L), high levels of hexanoic acid were detected in samples fermented by strains B4, B6, B7, B12, and B16. Octanoic acid was a major odorant of all samples (Supplementary Table S1) and can confer a “soapy” flavor to dairy products (Molimard and Spinnler, 1996; Güler, 2007). This carboxylic acid compound has been detected in fermented milk (Condursoa et al., 2008), goat milk cheese (Kim and Lindsay, 1991), and non-fat dry milk (Karagül-Yüceer et al., 2001). 3-Methylbutanoic acids are likely to originate from leucine. These branched-chain fatty acids are related to extensive breakdown of proteins (Kuzdzal-Savoie, 1980). 3-Methylbutanoic acids contribute to the aroma of fermented milk by conferring “rancid,” “sweaty,” and “cheesy” characteristics (Yvon and Rijnen, 2001). High concentrations of 3-methylbutanoic acids (2.6–103.5 μg/L) were found in nearly all samples in this study, except those fermented by strains B3 and B13. Similar results were found in cheddar cheese, as reported by Tammam et al. (2000). Although some major carboxylic acid compounds were detected in this study, they are not likely to contribute to fermented milk flavor due to their high perception threshold values.

Aldehyde compounds are important components of volatile flavor in dairy products. In all samples, acetaldehyde, 3-methyl-butanal, (E)-2-pentenal, hexanal, (E)-2-octenal, and nonanal were identified as key odorants (OAV > 1) of fermented milk due to their high concentrations and low aroma thresholds (Tables 3, 4). In particular, acetaldehyde and 3-methyl-butanal had OAVs of 1.04–8.28 and 0.33–3.33, respectively. Acetaldehyde is considered to contribute the typical flavor and aroma of yogurt, and to provide the “ethereal,” “fresh,” “green,” and “pungent” flavor to fermented milk (Panagiotidis et al., 2001; Gaafar, 2007). High levels of acetaldehyde (9.36–74.52 μg/L) were found in all samples in this study, except those fermented by strains B2, B8, and B15. This may be due to a lack of alcohol dehydrogenase enzyme in these strains, which is necessary for acetaldehyde conversion into ethanol (Chaves et al., 2002). 3-Methylbutanal is an important branched-chain aldehyde compound that contributes to good odor in dairy products (Madruga et al., 2009). This compound can be derived from leucine and contributes the pleasant aroma of fresh cheese to dairy products (Curioni and Bosset, 2002). 3-Methylbutanal can be converted into 3-methyl-butanol and 3-methyl-butanoic acid in dairy products (Afzal et al., 2012). In the present study, high levels of 3-methyl-butanoic acid (5.23–51.52 μg/L), 3-methylbutanal (1.76–15.63 μg/L), and 3-methyl-butanol (3.33–7.67 μg/L) were detected in all samples.

Ketone compounds are common components of most dairy products. Of all ketone compounds identified, 2,3-butanedione, acetoin, 2-heptanone, and 2-non-anone had OAVs > 1 (Table 3). 2,3-Butanedione is important for enhancing the flavor of dairy products (Gallardo-Escamilla et al., 2005). In this study, higher OAVs of 2,3-butanedione were found in samples fermented by strains B14, B16 and the control strain. 2,3-Butanedione contributes a buttery flavor to dairy products and can be converted to acetoin in the presence of the enzyme diacetyl reductase (Jo et al., 2018). As expected, acetoin (0.16–2.66) had an OAV > 1 in nearly all of our samples, except those fermented by strains B3, B12, and B13. OAVs for 2-heptanone and 2-octanone were higher in the sample fermented by strain B14 (30.35 and 13.10, respectively) than in the sample fermented by the control strain (20.58 and 11.93, respectively) (Table 3).

Alcohol compounds are considered to be the most important compounds with respect to the aroma of dairy products (Molimard and Spinnler, 1996). In this study, heptanol was the only alcohol compound with an OAV of >0.1; heptanol is a major flavor compound found in all samples (4.22–11.34 μg/L) (Cheng, 2010). 3-Methylbutanol can give an alcoholic, floral note to dairy products (Molimard and Spinnler, 1996). In this study, relatively large quantities (2.33–8.22 μg/L) of this compound were present in milk fermented by each of the 17 test strains of *L. delbrueckii* subsp. *bulgaricus*, and the control strain, upon completion of fermentation. In addition, some secondary alcohols were detected in this study, including 2-heptanol and 2-pentanol. 2-Heptanol was detected in samples fermented by strains B4, B7, B11, and B12 in the range of 1.16–1.44 μg/L. However, only trace concentrations of 2-pentanol were detected in the sample fermented by strain B15. This compound was detected by Dumont et al. (1974) in ripe Camembert cheeses.

The major pathways for the biosynthesis of esters in dairy systems involve two enzymatic mechanisms: esterification and alcoholysis (Liu et al., 2004). Formic acid ethenyl ester is considered to be an important flavor compound that imparts a pleasant fruity flavor to dairy products (Hong et al., 2018). The OAVs for formic acid ethenyl ester were 11.48, 10.11, 12.42, 7.82, and 1.4 in our samples fermented by strains B2, B5, B7, B14, and B17, which indicated that this compound could contribute significantly to the aroma of fermented milk.

Overall, 17 test strains of *L. delbrueckii* subsp. *bulgaricus* isolated from traditional fermented dairy products were used in this study. Among them, nine strains (B1, B3, B6, B9, B10, B11, B13, B14, and B16) had shorter fermentation times than the control strain (10 h). The fermentation and sensory characteristics of 17 test strains of *L. delbrueckii* subsp. *bulgaricus* during fermentation (3 h, 6 h) and storage (0 day, 12 h) were evaluated. Viable cell counts of samples fermented by strains B1, B2, B6, B8, B10, B11, B12, B14, and B16 approached 8.85 log_10_ CFU/mL (control strain sample) at the completion of fermentation, and cell viability was maintained during sample storage. The results of sensory assessment indicated that the flavor scores of samples B14 and B16 were more favorable than those of samples fermented by other strains. Furthermore, the results of dendrogram analysis indicated that the flavor profiles of samples fermented by strains B1, B4, B6, B8, B14, B15, B16, and B17 were more similar to that of the control strain. Based on fermentation characteristics, sensory assessment and flavor profiles, B14 and B16 offer good prospects for the production of fermented milk.

## Conclusion

This work demonstrated the influence of a variety of strains of *L. delbrueckii* subsp. *bulgaricus* when used as starter cultures for fermentation. Eighty-six volatile flavor compounds were identified in milk fermented by the 17 test strains of *L. delbrueckii* subsp. *bulgaricus*, and the control strain, upon completion of fermentation, including 17 carboxylic acids, 14 aldehydes, 13 ketones, 29 alcohols, 8 esters, and 5 aromatic hydrocarbon compounds. Some important volatile flavor compounds with OAVs > 1 were identified, including acetaldehyde, 3-methyl-butanal, (E)-2-pentenal, hexanal, (E)-2-octenal, nonanal, 2,3-butanedione, acetoin, 2-heptanone, 2-non-anone, and formic acid ethenyl ester. In addition, among the 17 test strains of *L. delbrueckii* subsp. *bulgaricus* in this study, strains B14 and B16 showed good fermentation characteristics in milk compared with those of the control strain. Nevertheless, further research is needed to ensure selection of *L. delbrueckii* subsp. *bulgaricus* strains with good flavor and fermentation characteristics, where such strains may serve as important resources in the development of fermented milk products.

## Data Availability Statement

All datasets generated for this study are included in the manuscript/the Supplementary Files.

## Author Contributions

TD and WL designed the experiments and drafted the manuscript. WR, YL, JT, and HC performed the experiments. All authors read and approved the final manuscript.

## Conflict of Interest Statement

The authors declare that the research was conducted in the absence of any commercial or financial relationships that could be construed as a potential conflict of interest.

## Supplementary Material

TABLE S1 Volatile compounds produced in milk fermented by 17 test strains of *L. delbrueckii* subsp. *bulgaricus*, and the control strain, upon completion of fermentation.
